# Trajectories of social health, cognitive, and daily functioning in community-dwelling older adults

**DOI:** 10.1093/geroni/igag010

**Published:** 2026-02-03

**Authors:** Anna Marseglia, Eline Verspoor, Marieke Perry, Myrra Vernooij-Dassen, Jeannie-Marie S Leoutsakos, Henry Brodaty, Jean Stafford, Mohammad Arfan Ikram, Joanna Rymaszewska, Anna-Karin Welmer, Karin Wolf-Ostermann, Frank J Wolters, Amaia Calderón-Larrañaga, René J F Melis, SHARED Consortium

**Affiliations:** Division of Clinical Geriatrics, Center for Alzheimer Research, Department of Neurobiology, Care Sciences, and Society, Karolinska Institutet, Stockholm, Sweden; Department of Geriatrics, Radboudumc Alzheimer Center, Radboud University Medical Center, Nijmegen, The Netherlands; Department of Geriatrics, Radboudumc Alzheimer Center, Radboud University Medical Center, Nijmegen, The Netherlands; Department of Primary and Community Care, Radboud University Medical Center, Nijmegen, The Netherlands; Department IQ Health, Radboud University Medical Center, Nijmegen, The Netherlands; Division of Geriatric Psychiatry and Neuropsychiatry, Department of Psychiatry and Behavioral Sciences, Johns Hopkins University School of Medicine, Baltimore, Maryland, United States; Department of Mental Health, Johns Hopkins Bloomberg School of Public Health, Baltimore, Maryland, United States; Centre for Healthy Brain Ageing (CHeBA), Discipline of Psychiatry and Mental Health, School of Clinical Medicine, University of New South Wales, Sydney, New South Wales, Australia; MRC Unit for Lifelong Health and Ageing at UCL, Faculty of Population Health Sciences, University College London, London, United Kingdom; Department of Epidemiology, Erasmus University Medical Center, Rotterdam, The Netherlands; Department of Clinical Neuroscience, Faculty of Medicine, Wroclaw University of Science and Technology, Wrocław, Poland; Aging Research Center, Department of Neurobiology, Care Sciences, and Society, Karolinska Institutet and Stockholm University, Stockholm, Sweden; Division of Physiotherapy, Department of Neurobiology, Care Sciences, and Society, Karolinska Institutet, Stockholm, Sweden; Institute for Public Health and Nursing Research (IPP), University of Bremen, Bremen, Germany; Department of Epidemiology, Erasmus University Medical Center, Rotterdam, The Netherlands; Department of Radiology and Nuclear Medicine, Erasmus MC – University Medical Center Rotterdam, Rotterdam, The Netherlands; Aging Research Center, Department of Neurobiology, Care Sciences, and Society, Karolinska Institutet and Stockholm University, Stockholm, Sweden; Stockholm Gerontology Research Center, Stockholm, Sweden; Department of Geriatrics, Radboudumc Alzheimer Center, Radboud University Medical Center, Nijmegen, The Netherlands

**Keywords:** Cognition, Daily functioning, Social health, Latent growth curve analysis, Latent class growth analysis, Cohort study

## Abstract

**Background and Objectives:**

Cognitive and functional impairments can both influence and result from deteriorating social health (SH), yet their interplay during aging remains poorly understood. This study investigated the concordance and discordance of trajectories in SH, cognitive, and daily functioning.

**Research Design and Methods:**

We analyzed 15-year follow-up data (2001–2015) from 2,848 initially dementia-free older adults in the Swedish National study on Aging and Care in Kungsholmen. Cognition and daily functioning were assessed with the Mini-Mental State Examination and activities of daily living (ADLs)/instrumental ADLs. SH encompassed indices of social participation, connections, and support. Trajectories across these five dimensions were identified using latent growth curve analyses, latent class growth analyses, and growth mixture models.

**Results:**

Two cognitive trajectories—relatively preserved (91%) and fast decline (9%)—and two daily functioning trajectories—stable (95%) and declining (5%)—were identified. SH trajectories included stable groups, gradually declining social participation (70%), and low initial social connections (29%). Social support showed stable (95%), declining (2%), and increasing (3%) trajectories. Women were more likely to belong to the initially low-stable social connections group, whereas higher education was linked to favorable trajectories across most dimensions but not social support. Concordance was observed among those with the lowest cognitive, daily functioning, and SH profiles. Notably, increasing social support was linked to low cognition but high daily functioning (odds ratios [OR] = 4.2, 95% CI: 2.3, 7.6).

**Discussion and Implications:**

Findings underscore the central role of SH in aging, particularly how dynamic changes in social participation, connections, and support relate to cognitive and functional outcomes.

Innovation and Translational Significance: Aging is marked by changes in cognitive and daily functioning, yet the role of dynamic social health changes in shaping these trajectories is overlooked. Using a data-driven approach, we identified distinct patterns across cognitive, functional, and social domains, revealing that increased social support is linked to better daily functioning, even when cognition declines. These findings suggest social health, particularly support, may play a key compensatory role. Targeting social resources may enhance functional independence in cognitively vulnerable older adults. This has implications for strategies and policies aiming to foster social engagement and support as modifiable levers to promote successful aging in the population.

## Background and objectives

Individuals do not age in isolation but within dynamic social environments. The concept of social health (SH) provides a valuable and novel framework for understanding how social factors—both at the individual and environmental levels—influence health and disease trajectories (Vernooij-Dassen et al., 2022). At the individual level, SH encompasses autonomy, fulfillment of social roles, and engagement in cognitively and physically stimulating activities. At the environmental level, SH includes the structure (e.g., frequency of relationships, network size/density/type), function (i.e., social network roles such as emotional and instrumental support), and appraisal (i.e., quality/satisfaction of social relationships, loneliness). These domains are illustrated in Supplementary Figure 1, depicting the SHARED consortium’s conceptual framework of SH (Vernooij-Dassen et al., 2022).

**Figure 1:**
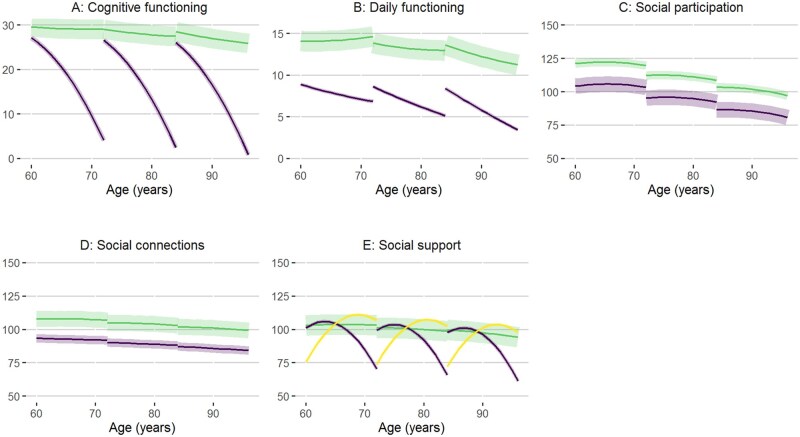
Growth models for the five outcomes (cognitive functioning in panel A, daily functioning in panel B, social participation in panel C, social connections in panel D, social support in panel E). The solid lines show the average trajectories for class 1 (green), class 2 (purple), and 3 (yellow, only for social support) by age at baseline (60–70 “sexagenarians,” 71–80 “septuagenarians,” and ≥81 years “octogenarians”). The shaded bands around the solid lines indicate the proportional size of the classes, when assigning participants to a class based on the most likely class: a wider shaded area indicates that a larger proportion of the population is assigned to that specific class.

Various indicators of poor SH have been associated with adverse health outcomes, including cognitive disorders, chronic diseases, depression, and mortality (Holt-Lunstad et al., 2010; Samtani et al., 2022; Triolo et al., 2022; Valtorta et al., 2016). However, most studies typically focused on single factors in isolation (e.g., social disengagement or loneliness), overlooking SH’s multidimensional nature and dynamic changes across the lifespan. These mid- to late-life changes in SH often occur alongside key shifts in cognitive and physical functioning (Saadeh et al., 2023). SH plays a crucial role, particularly as cognitive disorders emerge. Cognitive disorders can disrupt an individual’s engagement with their social environment, just as impoverished social environments can accelerate cognitive and overall health decline. Reduced engagement, fewer social contacts, and weak networks have all been associated with faster cognitive decline and increased dementia risk (Chen et al., 2025). The Lancet Commission estimated that 5% of global dementia cases are attributable to late-life social isolation (Livingston et al., 2024). Conversely, enhancing SH—e.g., promoting social participation—may support residual capacities, mitigating cognitive and functional declines (Mabire et al., 2018).

When cognition becomes impaired, individuals are less able to perform their daily lives. Functional impairment may also stem from, and/or contribute to, deterioration in SH: reduced independence can limit social interactions, while limited SH can, in turn, accelerate physical and functional declines. For instance, community-based studies have found strong associations of factors like separation/divorce, social isolation, reduced engagement in community activities, and feelings of loneliness with functional impairment, disability, or frailty (Connolly et al., 2017; Gale et al., 2018). Yet, the simultaneous evolution of SH, cognitive, and functional dimensions remains poorly understood. Uncovering simultaneous patterns of concordance and discordance across these dimensions could help identify profiles of vulnerability and resilience, informing targets for strategies that promote successful aging.

This study aimed to: (1) identify distinct subgroups (i.e., classes) of older adults based on changes in SH, cognitive functioning, and daily functioning; (2) assess the degree of alignment or divergence among these trajectories; and (3) investigate the sociodemographic and SH-related factors associated with both individual trajectories and subgroup patterns of concordance or discordance.

## Research design and methods

### Setting and study population

We used data from the Swedish National study on Aging and Care in Kungsholmen (SNAC-K), a community-based longitudinal cohort in Stockholm, initiated in 2001. An age-stratified random sample of 4,590 individuals, aged ≥60 years, living at home or in institutions, was invited to participate; 3,363 individuals were enrolled at baseline (March 2001–June 2024; 73% participation rate). Younger age cohorts (ages 60, 66, and 72) are followed every 6 years, and older age cohorts (≥78 years) every 3 years. At each wave, participants undergo comprehensive clinical and behavioral assessments conducted by trained physicians, nurses, and psychologists. Sociodemographic information (age, sex, education), SH, cognitive, and daily functioning are collected through structured interviews, questionnaires, and testing.

We analyzed data from the first five data-collection waves, up to 2015, including participants without baseline dementia who completed the Mini-Mental State Examination (MMSE) (*n *= 3,039). Excluded were participants with neuropsychiatric disorders (*n *= 40), living in nursing homes (*n *= 36), or with missing data on all SH variables (*n *= 115), yielding a final sample of 2,848 participants, followed over 15 years.

All waves of SNAC-K data collection were approved by the Regional Ethical Review Board in Stockholm or the Swedish Ethical Review Authority, with written informed consent obtained from all participants or their next of kin.

### Assessment of social health indicators

Details on interview protocols, questionnaires, and operationalizations of individual (social participation) and environmental (connections and support) SH indices have been previously published by Marseglia et al. (Supplementary Appendices A and B) (Marseglia et al., 2019) and are summarized below.

#### Social participation

At each wave, participants reported the frequency (0 = weekly, 1 = monthly, 2 = less frequently, 3 = never) of engaging in various leisure activities over the past year. Activities were categorized as predominantly mental (reading, painting/drawing/working with clay/pottery, playing chess/card games, playing a musical instrument, listening to music, using the internet/playing computer games), physical (gardening, hiking in the forest/picking berries or mushrooms, hunting/fishing, doing car/mechanical or home repairs, doing regular light-to-intense physical exercise), or social (attending cinema/theater/concerts, sport events, museums/art exhibitions, restaurants/bar/cafés, bingo, dancing, church service, travelling, volunteering, study circles/courses, and other social meetings) (Supplementary Appendix A). As activities span multiple dimensions, each was rated for its social, mental, and physical components, using a 4-point scale (0 = not-at-all to 3 = to-a-big-extent) by 78 raters (mean age 77 ± 7 years; 67% women; education 14.0 ± 4.7 years) (Kohncke et al., 2016).

To compute the social participation index, we (a) multiplied the activity frequency by its average social component weight, (b) summed the obtained scores across all activities, and (c) *z*-standardized the total score using the baseline mean and *SD* of cognitively unimpaired SNAC-K participants. Higher scores indicated greater social engagement. Follow-up scores were standardized using baseline values, and all *z*-scores were rescaled to a mean of 100 (*SD* 15) for easier interpretability of the growth models.

#### Social connections and support

Social connection index was derived based on the following items from the nurse interview and the Social Network Questionnaire (Supplementary Figure 1): relationship status (married, single, divorced, or widowed), living arrangement (living with someone, living alone), number of children as well as contact frequency (direct and remote) and network size (Supplementary Appendix B).

Social support index was derived based on the following items of the Social Network Questionnaire (Supplementary Appendix B): satisfaction with contacts, perceived material and psychological support, and sense of affinity.

Raw scores were *z*-standardized and averaged into the respective indices, where scores above 0 indicated higher connections or support compared to the study-population average (Marseglia et al., 2019). This procedure was repeated at follow-ups, using baseline values for *z*-standardization, and *z*-scores were rescaled to a mean of 100 (*SD* 15).

### Cognitive and daily functioning assessment

Global cognition was assessed using the MMSE (score range from 0 to 30), with higher scores reflecting better cognitive performance.

Daily functioning was assessed using the basic (ADL) and instrumental (IADL) activities of daily living scales. ADLs (score range 0–6) evaluate independence in personal care (bathing, dressing, toileting, continence, transferring/ambulating, eating), while IADLs (score range 0–8) assess more complex tasks (using the telephone, grocery shopping, food preparation, housekeeping, laundry, using public transportation, handling finances, taking medications) (Abbadi et al., 2024). Each item was scored one if the participant required assistance. ADL and IADL scores were combined (range 0–14) and reverse-coded so that higher scores indicated better daily functioning.

### Other variables

Age (years), sex (female versus male), and education (elementary, high school, university) were obtained at baseline via structured nurse interview. Dementia diagnosis followed the Diagnostic and Statistical Manual of Mental Disorders-4th edition and was independently made by an examining and a reviewing physician at each wave; disagreements were resolved by an external neurologist (Marseglia et al., 2019). For participants who died between visits, additional information was retrieved from medical records and the Swedish Cause of Death Register.

### Data analysis

For each of the five outcomes (social participation, connections, and support, cognitive, and daily functioning), a three-phase stepwise approach determined the optimal number of latent classes (Jung & Wickrama, 2008). Importantly, in each of these three steps, all growth parameters—the intercept, linear slope, and quadratic slope—were modeled in interaction with baseline chronological age. Baseline age (centered at 60 years) and its interaction with the SNAC-K wave were included to capture age-related variation in outcome trajectories.

First, one-class latent growth curve analyses (LGCA) assessed domain-specific changes over time (SNAC-K waves: 0 = baseline, 3, 6, 9, 12 years). Models tested both linear and quadratic change, as well as the inclusion of necessary random effects. Residual variances were either freely estimated or constrained across waves, depending on model fit. Next, latent class growth analyses (LCGA) were performed using the best-fitting LGCA model. Two- to four-class solutions were tested, both with and without random intercept. Finally, we fitted growth mixture models (GMM) with the identified number of classes through LCGA, progressively adding random effects: (a) overall random intercept, (b) overall random intercept, linear slope, and covariance, (c) class-specific random intercept, and (d) class-specific random intercept, linear slope, and covariance. Model fit was assessed via AIC, BIC, Lo-Mendel-Rubin test, entropy, class size, and class probability. Visual checks ensured the selection of the most parsimonious model. LCGA and GMM models used 500 random starts and 20 iterations.

Sociodemographic predictors of class membership (age, sex, education) were examined using a three-step approach for proximal variables, accounting for the fact that class membership is a latent rather than an observed variable (Vermunt, 2017).

To examine concordance across the five domain trajectories, we used a multiple pseudo-class drawing method to compute pairwise odds ratios (OR) between assigned growth classes. This method was also applied to compare sociodemographic and SH characteristics of participants with concordant versus discordant trajectories in cognition and functioning. This method accounts for latent class membership by multiply imputing the latent growth class using class membership probabilities. We imputed 20 times and performed logistic regressions for each imputation set that we combined using Rubin’s rule (Bray et al., 2015).

Data management was done in R. LGCA, LCGA, and GMM analyses were conducted using Mplus 8.4 and the R package MplusAutomation (Hallquist & Wiley, 2018). We followed the “Guidelines for Reporting on Latent Trajectory Studies” (van de Schoot et al., 2016).

## Results

### Baseline characteristics

Among the 2,848 participants (mean age 73 ± 10 years), the majority were female and highly educated (Table 1). Baseline cognitive and daily functioning were well preserved (median MMSE = 29; daily functioning = 14). Scores for SH indicators were above average among sexagenarians and below average among octogenarians. Participants’ characteristics across SNAC-K waves are shown in Supplementary Table 1.

**Table 1:** Baseline characteristics of the study participants by age group.

**Characteristics** ^a^	Overall	Sexagenarians	Septuagenarians	Octogenarians	*p* ^b^
	*N *= 2,848	*n *= 1,240	*n *= 864	*n *= 744	
**Age (years), mean (SD)**	73 (10)	63 (3)	75 (3)	87 (5)	<.001
**Female sex, *n* (%)**	1,781 (63%)	698 (56%)	552 (64%)	531 (71%)	<.001
**Education, *n* (%)**					
** Elementary**	1,006 (35%)	620 (50%)	254 (29%)	132 (18%)	<.001
** High school**	1,409 (49%)	530 (43%)	474 (55%)	405 (54%)	
** University**	433 (15%)	90 (7.3%)	136 (16%)	207 (28%)	
**MMSE score, median (IQR)**	29 (2.0)	30 (1)	29 (2)	28 (2)	<.001
**Daily functioning, median (IQR)**	14 (0)	14 (0)	14 (0)	14 (2)	<.001
**Social participation index, mean (SD)**	100 (15)	107 (12)	101 (14)	88 (15)	<.001
**Social connection index, mean (SD)**	100 (10)	102 (10)	100 (10)	96 (9)	<.001
**Social support index, mean (SD)**	100 (9)	102 (7)	100 (9)	97 (10)	<.001

*Note.* IQR = interquartile range; MMSE = Mini-Mental State Examination; *SD* = standard deviation.

a Missing data: MMSE = 4, daily functioning = 72, social connections = 65, social support = 64, social participation = 64.

b *p*-values were calculated using the chi-square test for categorical variables and one-way ANOVA or Kruskal–Wallis rank sum test for normally and non-normally distributed continuous variables, respectively.

### Trajectories of cognitive and daily functioning and SH

Two-class quadratic growth models best fit all domains, except social support, which required three classes (Figure 1). Model fit indices and diagnostic checks are shown in Supplementary Table 2; model selection details are shown in Supplementary Table 3.

**Table 2:** Associations between class membership for each outcome and sociodemographic factors.

Variables	Cognitive functioning		Daily functioning		Social participation		Social connections		Social support	
	OR (95% CI)	*p*	OR (95% CI)	*p*	OR (95% CI)	*p*	OR (95% CI)	*p*	OR (95% CI)	*p*
**Class 2 vs Class 1**										
** Baseline age (years, +1)**	1.11 (1.09, 1.13)	<.001	1.17 (1.13, 1.20)	<.001	0.99 (0.97, 1.00)	.010	0.99 (0.98, 1.01)	.330	1.03 (1.01, 1.06)	<.001
**Female (vs male)**	1.33 (0.90, 1.96)	.15	0.90 (0.59, 1.37)	.63	1.01 (0.80, 1.26)	.95	1.36 (1.08, 1.72)	.010	1.34 (0.72, 2.49)	.360
** Education**										
** Intermediate (vs lower)**	0.54 (0.38, 0.79)	<.001	0.65 (0.43, 1.00)	.05	0.31 (0.20, 0.50)	<.001	0.64 (0.47, 0.87)	<.001	0.85 (0.40, 1.78)	.660
** High (vs lower)**	0.30 (0.17, 0.52)	<.001	0.49 (0.27, 0.89)	.02	0.14 (0.09, 0.23)	<.001	0.31 (0.22, 0.44)	<.001	0.82 (0.34, 1.98)	.650
**Class 3 vs Class 1**										
** Baseline age (years, +1)**									1.08 (1.05, 1.11)	<.001
**Female (vs male)**									0.83 (0.51, 1.34)	.440
** Education**										
** Intermediate (vs lower)**									1.35 (0.78, 2.33)	.290
** High (vs lower)**									0.38 (0.16, 0.93)	.030

*Note*. CI = confidence interval; OR = odds ratio.

**Table 3:** Concordance across trajectories of cognitive and daily functioning, and social health.

Parameter	Cognitive functioning, class 2	Daily functioning, class 2	Social participation, class 2	Social connections, class 2
	OR (95% CI)	OR (95% CI)	OR (95% CI)	OR (95% CI)
**Cognitive functioning, class 2**				
**Daily functioning, class 2**	7.31 (4.80, 11.14)			
**Social participation, class 2**	2.38 (1.56, 3.62)	7.01 (2.89, 17.03)		
**Social connections, class 2**	1.85 (1.34, 2.55)	1.95 (1.27, 3.00)	2.93 (2.32, 3.70)	
**Social support, class 2**	2.31 (1.27, 4.19)	1.32 (0.46, 3.83)	1.99 (1.17, 3.38)	1.71 (1.09, 2.69)
**Social support, class 3**	4.52 (2.75, 7.42)	4.48 (2.38, 8.42)	3.39 (1.61, 7.12)	3.15 (1.96, 5.07)

*Note*. CI = confidence interval; OR = odds ratio.

For cognition, 91% of participants were assigned to class 1 with a high, relatively stable MMSE trajectory, and 9% to class 2 with an accelerated MMSE decline (Figure 1A; green and purple lines, respectively). Within classes, participants with a higher baseline age had a lower initial MMSE score (β = −0.045 MMSE points per year increase in baseline age) and an accelerated rate of decline (linear MMSE decline of −0.007/year and quadratic decline of −0.001/year^2^ in baseline age) (Supplementary Table 3).

For daily functioning, 95% of participants maintained relatively preserved daily functioning over time (class 1; green in Figure 1B), while 5% started with considerably impaired daily functioning (score of 8.9 [*SE* 0.28] for a 60-year-old individual) and declined steeply (linear decline of −0.21/year [*SE* 0.22] for a 60-year-old person) (class 2; purple in Figure 1B). Within classes, participants with a higher baseline age had lower initial daily functioning (β = −0.019 points per year increase in baseline age) and an accelerated rate of decline (linear daily functioning decline of −0.01/year and quadratic decline of −0.001/year^2^ in baseline age).

For social participation and connections (Figure 1C and D), both classes showed slowly declining trajectories over time. While for social participation the majority (70%) were in class 2, marked by poor initial engagement, decreasing over time (purple in Figure 1C), for social connections, the majority (71%) were in class 1 with initially richer connections (green in Figure 1D) than class 2. Baseline age was inversely associated with baseline participation and connections (β = −0.74 [*SE* 0.02] and β = −0.25 [*SE* 0.03], respectively) and linear and quadratic slope parameters (except for the linear slope in connections), indicating lower initial levels and faster declines of these SH indicators with increasing baseline age (Supplementary Appendix C).

For social support, 95% showed a stable support trajectory (class 1; green in Figure 1E), 2% exhibited declining support (class 2; purple in Figure 1E), and 3% increasing support (class 3; yellow in Figure 1E) over time.

Supplementary Figure 2 (top row) shows that the estimated random intercepts—reflecting the extent to which participants assigned to the specific class deviate at the start from the class-average trajectories—are particularly large for social connections and support, and smaller for cognitive and daily functioning. In contrast, the random linear slope variance—indicating deviations from the average slope within a class—is greater for daily functioning than cognition, social connections, and support.

### Sociodemographic determinants of class membership

Older baseline age was associated with greater odds of being in declining cognitive and daily functioning trajectories (class 2 vs class 1) and with declining (class 2 vs class 1) and increasing (class 3 vs class 1) social support trajectories (Table 2). Female participants had higher odds of being in social connections’ class 2, marked by poorer but stable connections (OR = 1.36, *p *≤ .01).

Higher education was associated with lower odds of faster cognitive and daily functioning declines (class 2 vs class 1) and with more favorable social participation and connections classes. Higher education was also linked to lower odds of being in the increasing social support class (OR = 0.38, *p *< .001).

### Concordance and discordance across cognitive functioning, daily functioning, and SH domains

Participants in the class with declining cognition were more likely to be in the daily functioning declining class (OR = 7.3 [95% CI: 4.8, 11.1]) (Table 3). Groups with poorer initial social participation or connections (class 2) were associated with both faster cognitive (OR = 2.3 [95% CI: 1.5, 3.6] and OR = 1.9 [95% CI: 1.3, 2.6], respectively) and functional (OR = 7.0 [95% CI: 2.9, 17.0] and OR = 2.0 [95% CI: 1.3, 3.1]) declines. Both decreasing and increasing social support trajectories were associated with higher odds of declining cognition. However, only decreasing support was linked to daily functioning decline (OR = 4.5 [95% CI: 2.4, 8.4]). Increasing and decreasing social support trajectories were also associated with poorer social participation and connection trajectories (class 2).

### Cognitive—daily functioning (dis)concordance and associated sociodemographic and SH factors

Most participants (*n *= 2,491, 88%) had stable cognitive and daily functioning trajectories (concordant “high cognitive & daily functioning”). A small minority (*n *= 47, 2%) declined in both (concordant “low cognitive & daily functioning”). Discordant patterns were observed in 3% of participants (*n *= 86) with stable cognition but declining daily functioning (discordant “high cognitive & low daily functioning”), and 7% (*n *= 194) with declining cognition but stable daily functioning (discordant “low cognitive & high daily functioning”).

The concordant “high cognitive & daily functioning” group (most optimal) was younger (mean age 71), had more men, and a higher proportion with university education (Table 4). Conversely, the concordant “low cognitive & daily functioning” group (least favorable) was the oldest (mean age 87) and had more women and individuals with elementary education. Social health profiles also differed markedly: favorable social support, participation, and connection trajectories (class 1) were more common in the concordant high-functioning group.

**Table 4:** Distribution of sociodemographic factors and social health indicators across four groups with concordance or discordance in class membership for cognitive and daily functioning trajectories.

Characteristics	Concordant		Discordant		*p* ^a^
	**High cognitive and daily functioning** *n *= 2,491 (88.4%)	**Low cognitive and daily functioning** *n *= 47 (1.7%)	**High cognitive and low daily functioning** *n *= 86 (3.0%)	**Low cognitive and high daily functioning** *n *= 194 (6.9%)	
**Age (years), mean (SD)**	71 (10)	87 (8)	86 (10)	81 (9)	<.001
**Sex, female, *n* (%)**	1,531 (61%)	39 (83%)	57 (66%)	143 (74%)	.003
**Education, *n* (%)**					
** Elementary**	314 (13%)	18 (38%)	29 (34%)	66 (34%)	<.001
** High school**	1,226 (49%)	23 (49%)	44 (51%)	100 (52%)	
** University**	951 (38%)	6 (13%)	13 (15%)	28 (14%)	
**MMSE score, median (IQR)**	29 (1)	25 (2)	28 (1)	26 (2)	<.001
**Daily functioning score, median (IQR)**	14 (1)	8 (2)	9 (2)	14 (1)	<.001
**Social participation index, mean (SD)**	102 (13)	72 (10)	77 (11)	90 (14)	<.001
**Social connection index, mean (SD)**	101 (10)	93 (9)	92 (9)	96 (10)	<.001
**Social support index, mean (SD)**	101 (8)	92 (12)	95 (11)	96 (11)	<.001
**Growth class**					
**Social participation, *n* (%)**					<.001
** 1 (stable)**	818 (33%)	1 (2%)	4 (5%)	36 (19%)	
** 2 (gradual decline)**	1,673 (67%)	46 (98%)	82 (95%)	158 (81%)	
**Social connections, *n* (%)**					<.001
** 1 (stable)**	1,815 (73%)	30 (64%)	48 (56%)	113 (58%)	
** 2 (initially lower, stable slope)**	676 (27%)	17 (36%)	38 (44%)	81 (42%)	
**Social support, *n* (%)**					<.001^b^
** 1 (stable)**	2,386 (96%)	40 (85%)	75 (87%)	164 (85%)	
** 2 (declining)**	45 (2%)	0 (0%)	1 (1%)	12 (6%)	
** 3 (increasing)**	60 (2%)	7 (15%)	10 (12%)	18 (9%)	

*Note.* IQR = interquartile range; MMSE = Mini-Mental State Examination; *SD* = standard deviation.

a Class characteristics for cognitive and daily functioning concordance are described by assigning participants to the class with the highest individual class probability. The pseudoclass membership method with 20 imputations was used to derive *p*-values from one-way ANOVAs or chi-square tests as appropriate, which were combined using Rubin’s rule.

b For cells with expected count below 5, *p*-values were calculated using Monte Carlo simulation with 2,000 replications via the “simulate.p.value()” function.

Nearly all participants with declining daily functioning—either with stable or declining cognition—belonged to the least favorable social participation trajectory (95%–98%). Among those with declining cognition, 19% with concomitant preserved daily functioning were assigned to the favorable social participation trajectory. This dropped to 2.1% when cognitive and daily functioning declines co-occurred.

For social support, participants with declining cognition but stable daily functioning belonged to the declining social support trajectory (6.2%). In contrast, none from the concordant “low cognitive & daily functioning” group was in the declining support trajectory, but 15% were in the increasing support trajectory, also observed in 12% of those with declining daily functioning but stable cognition.

Unfavorable participation (gradual decline) and connection (initially lower, stable decline) trajectories were associated with higher odds of unfavorable concordant and discordant cognitive and daily functioning (Supplementary Table 4). Similar results were observed for increasing and decreasing support for the cognitive-daily functioning discordant groups.

## Discussion and implications

In this large, population-based cohort of Swedish older adults, we identified distinct subgroups based on 15-year trajectories of cognitive functioning, daily functioning, and SH indicators—namely participation, connections, and support. Most participants (70%–95%) belonged to a class with relatively preserved function across all domains (class 1). A second group (class 2) showed steeper declines in cognition, daily functioning, and social support, alongside initially low but stable social connections and gradually decreasing participation. A third class emerged for social support alone (class 3), characterized by low initial support that increased over time, possibly reflecting enhanced resources from one’s social environment with age. Social participation was the only SH indicator for which the majority belonged to the less favorable class 2. While cognitive and daily functioning trajectories were often concordant, notable discordance was also observed. Particularly, participants with increasing social support over time were more likely to show discordant low cognitive functioning and high daily functioning trajectories, suggesting possible compensatory mechanisms.

Using growth mixture modeling enabled us to capture intraindividual changes over time in multiple, often connected outcomes, a key yet underutilized approach in aging research. Most prior studies focused on average trajectories of single health domains, thereby limiting cross-study comparisons. Our findings align with previous Swedish research showing stable social participation through late life, with decline typically occurring after age 70 (Finkel et al., 2018). This may reflect a shift with age toward choosing fewer but more meaningful activities, with efforts to maintain regular engagement (Saadeh et al., 2023). Similarly, U.S. GMM-based work identified largely stable patterns in social engagement over time, though one subgroup showed a faster decline rate—absent in our cohort (Thomas, 2011). Differences may stem from methodological (e.g., sample size, study design, follow-up length, social participation operationalization, statistical modeling), sociocultural, lifestyle, and/or intergenerational variations.

Few studies have examined trajectories of social connections or support in older age, with most focusing on frequency of social contacts and support at a single time point (Elovainio et al., 2018; Taylor & Lynch, 2004). Yet, prior SNAC-K findings suggest that high social connectedness and support may buffer against health decline in terms of multimorbidity burden, disability, and cognitive disorders (Calderón-Larrañaga et al., 2018; Dekhtyar et al., 2019)—results corroborated across other studies (Mogic et al., 2023).

We also found that sex and education influence cognitive, daily functioning, and SH trajectories. Women were more likely to follow a trajectory of stable social connections, consistent with evidence that, as they age, women sustain deeper, more stable ties and derive greater satisfaction from closer networks (Finkel et al., 2018). This may reflect both innate gender differences and historical generational roles. Higher education was consistently linked to more favorable trajectories across cognition, daily ­functioning, social participation, and social connections, likely ­reflecting cognitive reserve and/or broader socioeconomic advantages, such as greater access to resources and healthier behaviors (Fratiglioni et al., 2020). In turn, greater connectedness often supports better cognition, daily functioning, and social participation. Notably, individuals with poor cognitive and daily functioning were also represented in classes with declining or rising support in our study, emphasizing the dynamic nature of social support in responding to health changes.

Of note, while most participants showed declining social participation over time, those with sustained participation and stable support sometimes preserved daily functioning despite cognitive declines, suggesting a potential resilience pathway. This was especially evident among individuals with discordant patterns, that is, those maintaining daily functioning despite low cognition. These findings prompt future research into the factors that enable such resilience and how it might be fostered.

Participants with gradually declining social participation, low initial connections, and decreasing/increasing support trajectories were more likely to show discordant cognitive-daily functioning trajectories or concordantly low in both. As cognitive or physical abilities decline, social withdrawal may follow (Hill et al., 2022). However, some networks may respond by providing additional support. This aligns with our aforementioned observation that the discordant group (low cognition and high daily function) reported higher social participation than those low in both domains. Despite cognitive impairment, some individuals appear to maintain both physical independence and social engagement, underscoring a potentially adaptive subgroup that warrants closer investigation. Future research should aim at clarifying the mechanisms that enable this subgroup to retain autonomy despite cognitive decline.

Our findings also identify different factors associated with whether changes in cognitive and daily functioning trajectories were concordant or discordant. Specifically, the less favorable group (concordantly low) was associated with older age, female sex, and lower education, while the more optimal group (concordantly high) tended to be younger, predominantly male, with higher education and more favorable SH trajectories. Given the exploratory nature of this study, we refrain from drawing causal interpretations. Nonetheless, the association between concordantly low functioning and older age aligns with existing knowledge. Future studies should investigate why and how SH domains may influence simultaneously cognitive and physical trajectories—either accelerating decline or preserving function—and why these effects vary by sex and educational level.

### Strengths and limitations

Key strengths include a long follow-up period (15 years), comprehensive SH data that allowed us to implement a novel framework for a better understanding of SH dynamics in aging populations, and the use of GMM, which uncovers heterogeneous patterns not detectable by conventional models (e.g., mixed-effects models). A data-driven approach to subject classification improves predictions of late-life outcomes compared with average-level or a priori categorizations (Teas et al., 2023). However, we lacked computational power to run parallel process GMMs across domains, limiting our ability to analyze interdependencies among cognitive, functional, and SH trajectories. While theoretically feasible, parallel GMM models remain computationally intensive. Also, the highly educated sample may have biased the findings toward more favorable cognitive and daily functioning trajectories, possibly underestimating observed associations (Lövdén et al., 2020). Another particular limitation was the use of MMSE instead of a composite score from a cognitive battery. However, this was a carefully considered choice given participants’ advanced age, the long 15-year follow-up, the study’s focus on within-subject variability, and future cross-cohort validations in populations with diverse socio-cultural backgrounds. Indeed, older adults with cognitive disorders are more likely to drop out of complex cognitive battery assessments, as the tests become increasingly difficult with advancing impairment. This would have resulted in fewer participants and more biased trajectory effects. In contrast, MMSE is well-suited for capturing within-subject cognitive variability over time, particularly across repeated measures over a long follow-up period. Also, MMSE is widely used across different contexts, facilitating cross-cultural comparisons. Based on our findings, we hypothesize that high SH levels may buffer against health decline. However, it is also possible that high SH levels may instead result from stable good health (Calderón-Larrañaga et al., 2018; Gale et al., 2018). Lastly, we did not examine upstream determinants (e.g., lifestyle, multimorbidity, genetics) nor how mid- to late-life SH relates to later disability and cognitive disorders—key gaps that limit a full understanding of the potential bidirectional and synergistic links between SH, cognition, and daily functioning. Finally, while our findings may generalize to older adults with socio-cultural profiles similar to those of SNAC-K participants, replication in more diverse cohorts is needed.

## Conclusions

This study offers new insights into how trajectories of social participation, connections, and support evolve in later life, and how they interrelate with cognitive and physical functioning. While social participation and connections tend to remain stable or slowly decrease, social support shows greater variability, sometimes even increasing over time. Our findings suggest that stable SH, particularly support and participation, may preserve daily functioning even amid cognitive decline. Identifying the drivers enabling this favorable course is crucial toward informing future clinical and public health strategies aimed at maintaining independence and cognitive health in aging populations. While this exploratory study does not support direct policy recommendations, it lays the groundwork for hypothesis-driven research that may ultimately guide targeted interventions and, in turn, effective policy strategies in the future.

## Supplementary Material

igag010_Supplementary_Data

## Data Availability

Data are from the SNAC-K project, a population-based study that aims to increase our knowledge about the aging process from the physical, social, and mental perspectives (http://www.snac-k.se/). Access to the original data is available to the research community upon approval by the SNAC-K coordination group. Applications for accessing these data can be submitted via the website or through Maria Wahlberg (Maria.Wahlberg@ki.se) at the Aging Research Center, Karolinska Institutet. Code for data analyses is available on request from the corresponding author, Anna Marseglia (anna.marseglia@ki.se), and the PI of SNAC-K and senior author, Amaia Calderón-Larrañaga (amaia.calderon.larranaga@ki.se).
