# Supplementary material for: Trajectories of social health, cognitive, and daily functioning in community-dwelling older adults
Source: Innov Aging. 2026 Feb 3;10(5):igag010. doi: 10.1093/geroni/igag010 (PMC13147440; doi:10.1093/geroni/igag010)
Supplement: igag010_Supplementary_Data [file igag010_supplementary_data.docx]

***Innovation in Aging* Supplementary Material: Marseglia et al. (2026). Trajectories of social health, cognitive, and daily functioning in community-dwelling older adults.**

**TABLE OF CONTENTS**

| **Supplementary Figure 1** | Conceptual framework of Social Health (SH) as developed by the SHARED consortium and available markers in the SNAC-K cohort |
| --- | --- |
| **Appendix A** | Leisure activities interview protocol in SNAC-K |
| **Appendix B** | Social network questionnaire (Cornwell & Wait, 2009) |
| **Supplementary Table 1** | Characteristics of study participants at all study-visits, i.e., baseline (wave 1), 3-, 6-, 9- and 12-year follow-up (waves 2-5) |
| **Supplementary Table 2** | Fit statistics for the final Growth Mixture Models |
| **Appendix C** | Latent class growth analysis and growth mixture modeling: methodological details for class selection |
| **Supplementary Table 3** | The parameter estimates and class size by class for the final growth models for the five outcomes |
| **Supplementary Figure 2** | Estimated (top row) and observed (bottom row) trajectories by class for each of the five outcomes among 1000 randomly selected SNAC-K participants |
| **Supplementary Table 4** | Associations of social health indicators trajectories with cognitive and daily functioning concordance and discordance across four groups |

**Supplementary Figure 1**. Conceptual framework of Social Health (SH) as developed by the SHARED consortium and available markers in the SNAC-K cohort

The current study is grounded in the conceptual framework of SH developed by the SHARED consortium, established in 2019 under the EU-JPND program to advance understanding of how SH influence cognitive aging. SH markers—extending beyond isolation and loneliness—were identified and mapped through a systematic review of reviews (Lenart-Bugla et al 2022)). The SH framework conceptualizes SH as a dynamic interaction between individuals and their social environments. It includes: (a) Individual-level domains: reciprocity, autonomy, and social participation, and (b) Social-environmental domains: structure, function, and appraisal. Each domain can be operationalized through specific markers. In this figure, we highlight those available within the SNAC-K cohort.

*Reciprocity and stigma are conceptually important but currently lack direct or proxy measures in SNAC-K.

**References**

Lenart-Bugla, M., Łuc, M., Pawłowski, M., Szcześniak, D., Seifert, I., Wiegelmann, H. (2022). What do we know about scial and non-social factors influencing the pathway from cognitive health to dementia? A Systematic review of reviews. Brain sciences, 12, 1214.

**APPENDIX A.** Leisure activities interview protocol in SNAC-K

Instructions: Following are questions regarding your leisure activities. Write or check the answer that best suits you.

| Have you participated in any of the following entertainment or cultural activities in the last 12 months? | |
| --- | --- |
| 1. Cinema/Theatre/Concert | For each item:  Yes, to the same degree  Yes, to a higher degree  Yes, but to a lesser degree  No  No response |
| 1. Sporting Events |  |
| 1. Museum/Art exhibit |  |
| 1. Go to Restaurant/Pub/Café |  |
| 1. Bingo |  |
| 1. Dancing |  |
| 1. Attend church/revival meeting |  |
| 1. Participate in study circle or a course |  |
| 1. Participate in volunteer work |  |
| 1. Participate in association/club work |  |
| 1. Travel |  |
| 1. Gardening |  |
| 1. Hiking in forest/picking berries, mushrooms |  |
| 1. Hunting, fishing |  |
| 1. Knitting, weaving, sewing |  |
| 1. Painting, drawing, working with clay/pottery |  |
| 1. Home repairs |  |
| 1. Car or mechanical repairs |  |
| 1. Read the newspaper/ magazine or journal/books |  |
| 1. Watch TV |  |
| 1. Play chess/card games |  |
| 1. Play musical instrument |  |
| 1. Listen to music |  |
| 1. Use the internet or play computer games |  |

| In the last 12 months: | |
| --- | --- |
| 1. Did you regularly engage in light exercise (e.g. walking along roads or in parks, walking in the woods, short bicycle rides, light aerobics, golf)? | Every day; Several times a week; 2-3 times/month; Less; Never; Don't know; No response; Several options checked |
| 1. Did you regularly engage in moderate to intense exercise, now or previously (e.g. jogging, long power walks, heavy-duty gardening, long bicycle rides, high-intensity aerobics, long distance ice skating, swimming, ball sports (not golf) or other similar activity)? | Every day; Several times a week; 2-3 times/month; Less; Never; Don't know; No response; Several options checked |

**APPENDIX B.** Social network questionnaire

*Reference: Cornwell, E. Y., & Waite, L. J. (2009). Measuring social isolation among older adults using multiple indicators from the NSHAP study. The journals of gerontology. Series B, Psychological sciences and social sciences, 64 Suppl 1(Suppl 1), i38–i46. https://doi.org/10.1093/geronb/gbp037*

Instructions: Following there are questions regarding your social network. Write or check the answers that best suit you.

*Frequency of direct or remote contacts*

| How often do you meet the following in person? | |
| --- | --- |
| 1. Parents | For each item:  Daily, more than twice/week  Weekly, more than twice/month  Monthly, more than 6 times/year  Quarterly, more than once/year  Less often  Never  Don't know  N/A (e.g., have no children or parent no longer alive)  No response  Several options checked |
| 1. Children |  |
| 1. Son/daughter-in-law |  |
| 1. Grandchildren |  |
| 1. Siblings |  |
| 1. Other relative |  |
| 1. Neighbour |  |
| 1. Friend |  |

| How often are you in touch, via telephone, letters, e-mail with the following: | |
| --- | --- |
| 1. Parents | For each item:  Daily, more than twice/week  Weekly, more than twice/month  Monthly, more than 6 times/year  Quarterly, more than once/year  Less often  Never  Don't know  N/A (e.g., have no children or parent no longer alive)  No response  Several options checked |
| 1. Children |  |
| 1. Son/daughter-in-law |  |
| 1. Grandchildren |  |
| 1. Siblings |  |
| 1. Other relative |  |
| 1. Neighbour |  |
| 1. Friend |  |

*Satisfaction with these contacts*

| Are you satisfied with these contacts? | |
| --- | --- |
| 1. Parents | For each item:  Yes  No  Don't know  N/A (e.g. do not have children or parents no longer alive)  No response  Several options checked |
| 1. Children |  |
| 1. Son/Daughter-in-law |  |
| 1. Grandchildren |  |
| 1. Siblings |  |
| 1. Other relative |  |
| 1. Neighbour |  |
| 1. Friend |  |

*Perceived material support*

| 1. Could you get help from one or several people in case of illness or other practical troubles? (e.g. borrow little things, get help with repairs, get advice and information) | For each item:  Yes, without a doubt  Yes, probably  No, probably not  No, not at all  Don't know  No response  Several options checked |
| --- | --- |
| 1. Do you know one or several people who could help you write an official letter or appeal a government decision? |  |

*Social network size*

| 1. How many people do you feel you know well and can talk to about most things? (e.g. relatives, friends, neighbours, and/or colleagues) | None; 1-2 people; 3 people; 4-6 people; 7-9 people; 10-15 people; 16-30 people; More than 30 people; Don't know; No response; Several options checked |
| --- | --- |

*Perceived psychological support*

| 1. Do you feel that you know one or a few people who could give you proper personal/emotional support to manage the stress and troubles of life? | For each item:  Yes, without a doubt  Yes, probably  No, probably not  No, not at all  Don't know  No response  Several options checked |
| --- | --- |
| 1. Do you know someone with whom you can be yourself, who accepts you for all your good and bad qualities? |  |

*Sense of affinity with association members*

| 1. If you are part of an association/organization, would you say you feel a strong sense of belonging to this group and its members? | I am not member of any association; To a high degree; To a modest degree; Not especially; Not at all; Don't know; No response; Several options checked |
| --- | --- |

*Sense of affinity with relatives*

| 1. Do you feel a strong sense of kinship with your relatives (beyond spouse/partner/children)? | Have no family; To a high degree; To a modest degree; Not especially; Not at all; Don't know; No response; Several options checked |
| --- | --- |

*Sense of affinity with residence area*

| 1. Are you rooted in and feel a strong sense of kinship with your neighbourhood? | To a high degree; To a certain degree; Not especially; Not at all; Don't know; No response; Several options checked |
| --- | --- |

*Being part of a group of friends*

| 1. Are you part of a group of friends/acquaintances who have something in common or do some activity together (e.g., play cards, listen to music, go on excursions, etc.)? | Yes; No; Don't know; No response; Several options checked |
| --- | --- |

**Supplementary Table 1.** Characteristics of study participants at all study-visits, i.e., baseline (wave 1), 3-, 6-, 9-, and 12-year follow-up (waves 2-5)

|  | **Followed**  **in W1 W3 W5** | | | **Followed in**  **W1 W3 W4 W5** | | | | **Followed in**  **W1 W2 W3 W4 W5** | | | | |
| --- | --- | --- | --- | --- | --- | --- | --- | --- | --- | --- | --- | --- |
| **Characteristic** | **W1**  n=1,240 | **W3**  n=1,022 | **W5**  n=835 | **W1**  n=442 | **W3**  n=340 | **W4**  n=275 | **W5**  n=221 | **W1**  n=1,166 | **W2**  n=847 | **W3**  n=602 | **W4**  n=403 | **W5**  n=222 |
| **Age at wave (years)** | 63.0 (2.9) | 68.7 (2.9) | 74.4 (2.9) | 72.3 (0.2) | 78.1 (0.2) | 81.0 (0.3) | 84.0 (0.2) | 83.6 (5.4) | 86.0 (5.0) | 87.8 (4.4) | 90.0 (3.9) | 92.1 (3.1) |
| **Age group at W1** |  |  |  |  |  |  |  |  |  |  |  |  |
| Sexagenarians | 1,240 (100%) | 1,022 (100%) | 835 (100%) | 0 (0%) | 0 (0%) | 0 (0%) | 0 (0%) | 0 (0%) | 0 (0%) | 0 (0%) | 0 (0%) | 0 (0%) |
| Septuagenarians | 0 (0%) | 0 (0%) | 0 (0%) | 442 (100%) | 340 (100%) | 275 (100%) | 221 (100%) | 422 (36%) | 333 (39%) | 278 (46%) | 206 (51%) | 131 (59%) |
| Octogenarians | 0 (0%) | 0 (0%) | 0 (0%) | 0 (0%) | 0 (0%) | 0 (0%) | 0 (0%) | 744 (64%) | 514 (61%) | 324 (54%) | 197 (49%) | 91 (41%) |
| **Sex, female, *n* (%)** | 698 (56%) | 589 (58%) | 495 (59%) | 265 (60%) | 214 (63%) | 171 (62%) | 144 (65%) | 818 (70%) | 602 (71%) | 424 (70%) | 292 (72%) | 167 (75%) |
| **Education, n (%)** |  |  |  |  |  |  |  |  |  |  |  |  |
| Higher | 620 (50%) | 528 (52%) | 448 (54%) | 150 (34%) | 117 (34%) | 100 (36%) | 82 (37%) | 236 (20%) | 184 (22%) | 142 (24%) | 97 (24%) | 54 (24%) |
| Intermediate | 530 (43%) | 433 (42%) | 342 (41%) | 239 (54%) | 181 (53%) | 143 (52%) | 113 (51%) | 640 (55%) | 472 (56%) | 331 (55%) | 224 (56%) | 126 (57%) |
| Lower | 90 (7.3%) | 61 (6.0%) | 45 (5.4%) | 53 (12%) | 42 (12%) | 32 (12%) | 26 (12%) | 290 (25%) | 191 (23%) | 129 (21%) | 82 (20%) | 42 (19%) |
| **Cognitive functioning, MMSE score, median (IQR)** | 30.0 (1.0) | 29.0 (2.0) | 29.0 (2.0) | 29.0 (1.0) | 28.0 (2.0) | 28.0 (3.0) | 28.0 (3.0) | 29.0 (2.0) | 28.0 (3.0) | 27.0 (4.0) | 27.0 (5.0) | 26.0 (6.0) |
| **Daily functioning, score, median (IQR)** | 14.0 (0.0) | 14.0 (0.0) | 14.0 (0.0) | 14.0 (0.0) | 14.0 (1.0) | 14.0 (1.0) | 14.0 (1.0) | 14.0 (1.0) | 13.0 (1.0) | 13.0 (4.0) | 12.0 (6.0) | 11.0 (6.0) |
| **Social participation, index, mean (SD)** | 106.6 (11.7) | 108.8 (10.8) | 106.2 (11.8) | 102.8 (12.8) | 101.2 (12.4) | 99.9 (12.1) | 97.3 (13.5) | 92.1 (15.1) | 93.6 (13.0) | 92.4 (12.4) | 90.6 (11.7) | 86.7 (14.4) |
| **Social connections, index, mean (SD)** | 102.3 (9.9) | 102.6 (9.8) | 101.9 (9.7) | 101.6 (9.6) | 100.7 (8.9) | 100.4 (8.8) | 98.8 (8.7) | 97.0 (9.5) | 97.2 (9.5) | 97.5 (9.2) | 96.8 (8.9) | 93.2 (10.0) |
| **Social support, index, mean (SD)** | 101.9 (7.4) | 103.0 (6.8) | 102.1 (8.0) | 100.8 (8.4) | 100.9 (8.3) | 101.0 (8.2) | 98.8 (10.8) | 97.8 (9.8) | 98.5 (9.4) | 99.3 (9.2) | 99.0 (9.3) | 93.7 (15.0) |

Data are presented as mean (SD), n (%), or median (IQR).

Abbreviations: MMSE, Mini-Mental State Examination; IQR, interquartile range; SD, standard deviations; W1-5, examination wave 1 to 5.

**Supplementary Table 2.** Fit statistics for the final Growth Mixture Models

|  | **N class** | **Parameters** | **Class_counts** | **Class_props** | **LL** | **AIC** | **BIC** | **Entropy** |
| --- | --- | --- | --- | --- | --- | --- | --- | --- |
| **Cognitive functioning** - Quadratic growth, overall random intercept & linear slope, overall adjustment for BL age, free residuals | | | | | | | | |
| Final model | 2 | 18 | 2599 247 | 0.91 0.09 | -16,456.58 | 32949.16 | 33056.32 | 0.914 |
| First replication using OPTSEED | 2 | 18 | 2599 247 | 0.91 0.09 | -16,456.58 | 32949.16 | 33056.32 | 0.914 |
| Second replication using OPTSEED | 2 | 18 | 2599 247 | 0.91 0.09 | -16,456.58 | 32949.16 | 33056.32 | 0.914 |
| **Daily functioning** - Quadratic growth, overall random intercept & linear slope, overall adjustment for BL age, free residuals | | | | | | | | |
| Final model | 2 | 18 | 2687 133 | 0.95 0.05 | -12,596.91 | 25229.82 | 25336.82 | 0.994 |
| First replication using OPTSEED | 2 | 18 | 2687 133 | 0.95 0.05 | -12,596.91 | 25229.82 | 25336.82 | 0.994 |
| Second replication using OPTSEED | 2 | 18 | 133 2687 | 0.05 0.95 | -12,596.91 | 25229.82 | 25336.82 | 0.994 |
| **Social connections** - Quadratic growth, class specific random intercept, overall adjustment for BL age, free residuals | | | | | | | | |
| Final model | 2 | 19 | 828 2020 | 0.29 0.71 | -22,117.17 | 44272.34 | 44385.47 | 0.641 |
| First replication using OPTSEED | 2 | 19 | 828 2020 | 0.29 0.71 | -22,117.17 | 44272.34 | 44385.47 | 0.641 |
| Second replication using OPTSEED | 2 | 19 | 2020 828 | 0.71 0.29 | -22,117.17 | 44272.34 | 44385.47 | 0.641 |
| **Social support** - Quadratic growth, overall random intercept & linear slope, overall adjustment for BL age, free residuals | | | | | | | | |
| Final model | 3 | 22 | 2693 58 97 | 0.95 0.02 0.03 | -22,789.90 | 45623.80 | 45754.79 | 0.889 |
| First replication using OPTSEED | 3 | 22 | 2693 58 97 | 0.95 0.02 0.03 | -22,789.90 | 45623.80 | 45754.79 | 0.889 |
| Second replication using OPTSEED | 3 | 22 | 2693 58 97 | 0.95 0.02 0.03 | -22,789.90 | 45623.80 | 45754.79 | 0.889 |
| **Social participation** - Quadratic growth, no random effects, overall adjustment for BL age, free residuals | | | | | | | | |
| Final model | 2 | 15 | 1987 861 | 0.7 0.3 | -25,754.17 | 51538.34 | 51627.65 | 0.658 |
| First replication using OPTSEED | 2 | 15 | 1987 861 | 0.7 0.3 | -25,754.17 | 51538.34 | 51627.65 | 0.658 |
| Second replication using OPTSEED | 2 | 15 | 1987 861 | 0.7 0.3 | -25,754.17 | 51538.34 | 51627.65 | 0.658 |

**Appendix C. Latent class growth analysis and growth mixture modeling: methodological details for class selection**

We followed Jung and Wickrama’s model-building strategy for latent class growth analysis and growth mixture modeling (Jung & Wickrama 2008).

**The figure below** shows the Bayesian information criteria (BIC) values plotted against the number of parameters in each model for the three steps of the model building strategy:

- Latent Growth Curve Analysis (LGCA): Yellow, squares, identifying growth type (linear or quadratic);
- Latent Class Growth Analysis (LCGA): Purple, triangles, identifying the number of growth classes (1 to 4);
- Growth Mixture Modeling (GMM): Green, circles, identifying the random effects needed to further improve fit.

The number indicates the number of classes.


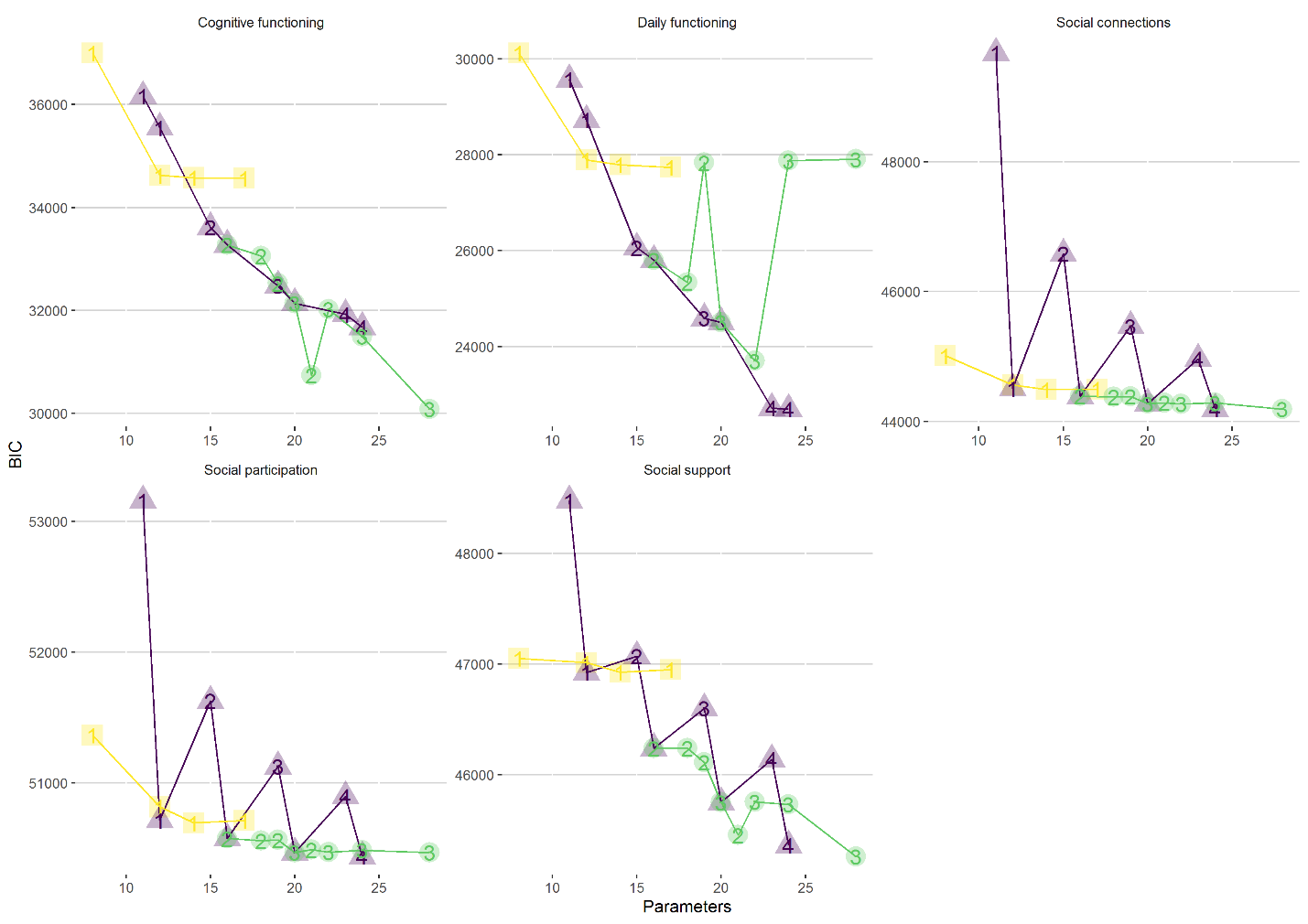


For **LGCA**, we fitted models in the following order (yellow squares):

- - Means model with fixed and random intercept, adjusted for baseline age, with free residuals for each time point;
  - Linear growth model with fixed and random intercept and linear slope, adjusted for baseline, with free residuals for each time point;
  - Quadratic growth model with fixed intercept, linear and quadratic slope and random intercept and linear slope, adjusted for baseline age, with free residuals for each time point;
  - Quadratic growth model with fixed intercept, linear and quadratic slope and random intercept and linear slope, adjusted for baseline age, with free residuals for each time point;
  - Quadratic growth model with fixed and random intercept, linear and quadratic slope, adjusted for baseline age, with free residuals for each time point.

For **LCGA**, we fitted models in the following order (purple triangles):

- 1 to 4 class model with fixed intercept, linear and quadratic slope without any random effects, adjusted for baseline age, with free residuals for each time point
- 1 to 4 class model with fixed intercept, linear and quadratic slope WITH an overall random intercept, adjusted for baseline age, with free residuals for each time point (essentially this is a GMM and not an LCGA model, as the model includes a random effect.

For **GMM** (green circles), we fitted 2 and 3 class models with fixed intercept, linear and quadratic slope and, in order:

- Overall random intercept
- Overall random intercept and linear slope
- Class specific random intercept
- Class specific random intercept and linear slope

**Key findings:**

- While the addition of (linear) growth factors considerably improved model fit indicated by a decline in BIC in the subsequent LGCA models (yellow line) for cognitive and daily functioning, this was less prominent in particularly social connections and social participation
- While an additional class (LCGA and GMM) resulted in a continued decline in BIC for cognitive functioning, this effect was less prominent for the three social outcomes.
- In contrast, while the addition of a random intercept to the model resulted in a huge decline in BIC for particularly social participation and social connections (compare e.g., the BIC for the two 1-class LCGA models (in green)), this effect is much less prominent in cognitive and daily functioning (compare - for cognitive and daily functioning – the difference in BIC for the two 1-class LCGA models (one without random intercept vs one WITH random intercept) against the change in BIC when adding a second class).
- The resulting improvement in model fit with increasing model complexity in the GMM step is shallower in social participation and connections than in cognitive and daily functioning.
- The increases in BIC (i.e. decrease in model fit) for some daily functioning models of the GMM step indicated overfitted models. The most complex GMM model for daily functioning did not converge, which is why no estimate of BIC is provided for this model.

**Supplementary Table 3.** The parameter estimates and class size by class for the final growth models for the five outcomes.

| **Parameter** | **Class** | **Cognitive functioning** | | **Daily functioning** | | | **Social participation** | | | **Social connections** | | | **Social support** | | |  |
| --- | --- | --- | --- | --- | --- | --- | --- | --- | --- | --- | --- | --- | --- | --- | --- | --- |
|  |  | **estimate (SE)** | ***p*** | | **estimate (SE)** | ***p*** | | **estimate (SE)** | ***p*** | | **estimate (SE)** | ***p*** | | **estimate (SE)** | ***p*** | |
| **Class 1** |  |  |  | |  |  | |  |  | |  |  | |  |  | |
| **Fixed intercept** | 1 | 29.57 (0.032) | <0.001 | | 14.063 (0.02) | <0.001 | | 121.016 (0.846) | <0.001 | | 107.606 (0.679) | <0.001 | | 102.874 (0.212) | <0.001 | |
| Baseline age (+1 year) * Fixed intercept | 1 | -0.045 (0.003) | <0.001 | | -0.019 (0.002) | <0.001 | | -0.74 (0.027) | <0.001 | | -0.251 (0.018) | <0.001 | | -0.142 (0.014) | <0.001 | |
| **Fixed linear slope** | 1 | -0.109 (0.019) | <0.001 | | -0.018 (0.01) | 0.108 | | 0.455 (0.133) | 0.001 | | 0.136 (0.064) | 0.033 | | 0.213 (0.065) | 0.001 | |
| Baseline age (+1 year) * Fixed linear slope | 1 | -0.007 (0.002) | 0.001 | | -0.01 (0.002) | <0.001 | | -0.017 (0.008) | 0.030 | | -0.005 (0.004) | 0.194 | | -0.019 (0.006) | 0.002 | |
| **Fixed quadratic slope** | 1 | 0.006 (0.002) | <0.001 | | 0.007 (0.001) | <0.001 | | -0.045 (0.011) | <0.001 | | -0.016 (0.005) | 0.002 | | -0.017 (0.005) | 0.002 | |
| Baseline age (+1 year) * Fixed quadratic slope | 1 | -0.001 (0.000) | 0.016 | | -0.001 (0.000) | <0.001 | | -0.002 (0.001) | 0.004 | | -0.002 (0.000) | <0.001 | | 0.001 (0.001) | 0.338 | |
| Random intercept variance | 1 | 0.499 |  | | 0.234 |  | |  |  | | 29.28 |  | | 32.526 |  | |
| Covariance RI and RL | 1 | 0.065 |  | | 0.061 |  | |  |  | | -0.603 |  | | 0.17 |  | |
| Random linear slope variance | 1 | 0.026 |  | | 0.066 |  | |  |  | | 0.086 |  | | 0.029 |  | |
| Random quadratic slope variance | 1 |  |  | |  |  | |  |  | |  |  | |  |  | |
| Class size, *n* (%) | 1 | 2599 (0.91) |  | | 2687 (0.95) |  | | 861 (0.3) |  | | 2020 (0.71) |  | | 2693 (0.95) |  | |
| **Class 2** |  |  |  | |  |  | |  |  | |  |  | |  |  | |
| **Fixed intercept** | 2 | 27.11 (0.206) | <0.001 | | 8.865 (0.280) | <0.001 | | 104.091 (0.594) | <0.001 | | 93.213 (1.037) | <0.001 | | 101.02 (1.506) | <0.001 | |
| Baseline age (+1 year) * Fixed intercept | 2 | -0.045 (0.003) | <0.001 | | -0.019 (0.002) | <0.001 | | -0.74 (0.027) | <0.001 | | -0.251 (0.018) | <0.001 | | -0.142 (0.014) | <0.001 | |
| **Fixed linear slope** | 2 | -0.883 (0.255) | 0.001 | | -0.214 (0.215) | 0.318 | | 0.567 (0.115) | <0.001 | | -0.125 (0.112) | 0.267 | | 3.015 (0.532) | <0.001 | |
| Baseline age (+1 year) * Fixed linear slope | 2 | -0.007 (0.002) | 0.001 | | -0.01 (0.002) | <0.001 | | -0.017 (0.008) | 0.03 | | -0.005 (0.004) | 0.194 | | -0.019 (0.006) | 0.002 | |
| **Fixed quadratic slope** | 2 | -0.086 (0.021) | <0.001 | | 0.005 (0.022) | 0.829 | | -0.05 (0.009) | <0.001 | | 0.002 (0.009) | 0.803 | | -0.465 (0.044) | <0.001 | |
| Baseline age (+1 year) * Fixed quadratic slope | 2 | -0.001 (0.000) | 0.016 | | -0.001 (0.000) | <0.001 | | -0.002 (0.001) | 0.004 | | -0.002 (0.000) | <0.001 | | 0.001 (0.001) | 0.338 | |
| Random intercept variance | 2 | 0.499 |  | | 0.234 |  | |  |  | | 38.045 |  | | 32.526 |  | |
| Covariance RI and RL | 2 | 0.065 |  | | 0.061 |  | |  |  | | -0.603 |  | | 0.17 |  | |
| Random linear slope variance | 2 | 0.026 |  | | 0.066 |  | |  |  | | 0.086 |  | | 0.029 |  | |
| Random quadratic slope variance | 2 |  |  | |  |  | |  |  | |  |  | |  |  | |
| Class size, *n* (%) | 2 | 247 (0.09) |  | | 133 (0.05) |  | | 1987 (0.7) |  | | 828 (0.29) |  | | 58 (0.02) |  | |
| **Class 3** |  |  |  | |  |  | |  |  | |  |  | |  |  | |
| **Fixed intercept** | 3 |  |  | |  |  | |  |  | |  |  | | 75.425 (0.952) | <0.001 | |
| Baseline age (+1 year) * Fixed intercept | 3 |  |  | |  |  | |  |  | |  |  | | -0.142 (0.014) | <0.001 | |
| **Fixed linear slope** | 3 |  |  | |  |  | |  |  | |  |  | | 7.985 (0.866) | <0.001 | |
| Baseline age (+1 year) * Fixed linear slope | 3 |  |  | |  |  | |  |  | |  |  | | -0.019 (0.006) | 0.002 | |
| **Fixed quadratic slope** | 3 |  |  | |  |  | |  |  | |  |  | | -0.449 (0.067) | <0.001 | |
| Baseline age (+1 year) * Fixed quadratic slope | 3 |  |  | |  |  | |  |  | |  |  | | 0.001 (0.001) | 0.338 | |
| Random intercept variance | 3 |  |  | |  |  | |  |  | |  |  | | 32.526 |  | |
| Covariance RI and RL | 3 |  |  | |  |  | |  |  | |  |  | | 0.17 |  | |
| Random linear slope variance | 3 |  |  | |  |  | |  |  | |  |  | | 0.029 |  | |
| Random quadratic slope variance | 3 |  |  | |  |  | |  |  | |  |  | |  |  | |
| Class size, *n* (%) | 3 |  |  | |  |  | |  |  | |  |  | | 97 (0.03) |  | |
|  |  |  |  | |  |  | |  |  | |  |  | |  |  | |
| Residual error time point 1 variance | any | 0.922 |  | | 0.208 |  | | 102.292 |  | | 13.007 |  | | 16.566 |  | |
| Residual error time point 2 variance | any | 9.727 |  | | 2.278 |  | | 71.553 |  | | 13.443 |  | | 42.993 |  | |
| Residual error time point 3 variance | any | 3.05 |  | | 1.084 |  | | 67.804 |  | | 9.698 |  | | 26.3 |  | |
| Residual error time point 4 variance | any | 10.603 |  | | 4.656 |  | | 70.526 |  | | 11.915 |  | | 28.824 |  | |
| Residual error time point 5 variance | any | 2.917 |  | | 1.421 |  | | 86.983 |  | | 16.05 |  | | 13.954 |  | |

**Supplementary Figure 2.** Estimated (top row) and observed (bottom row) trajectories by class for each of the five outcomes among 1000 randomly selected SNAC-K participants.

**
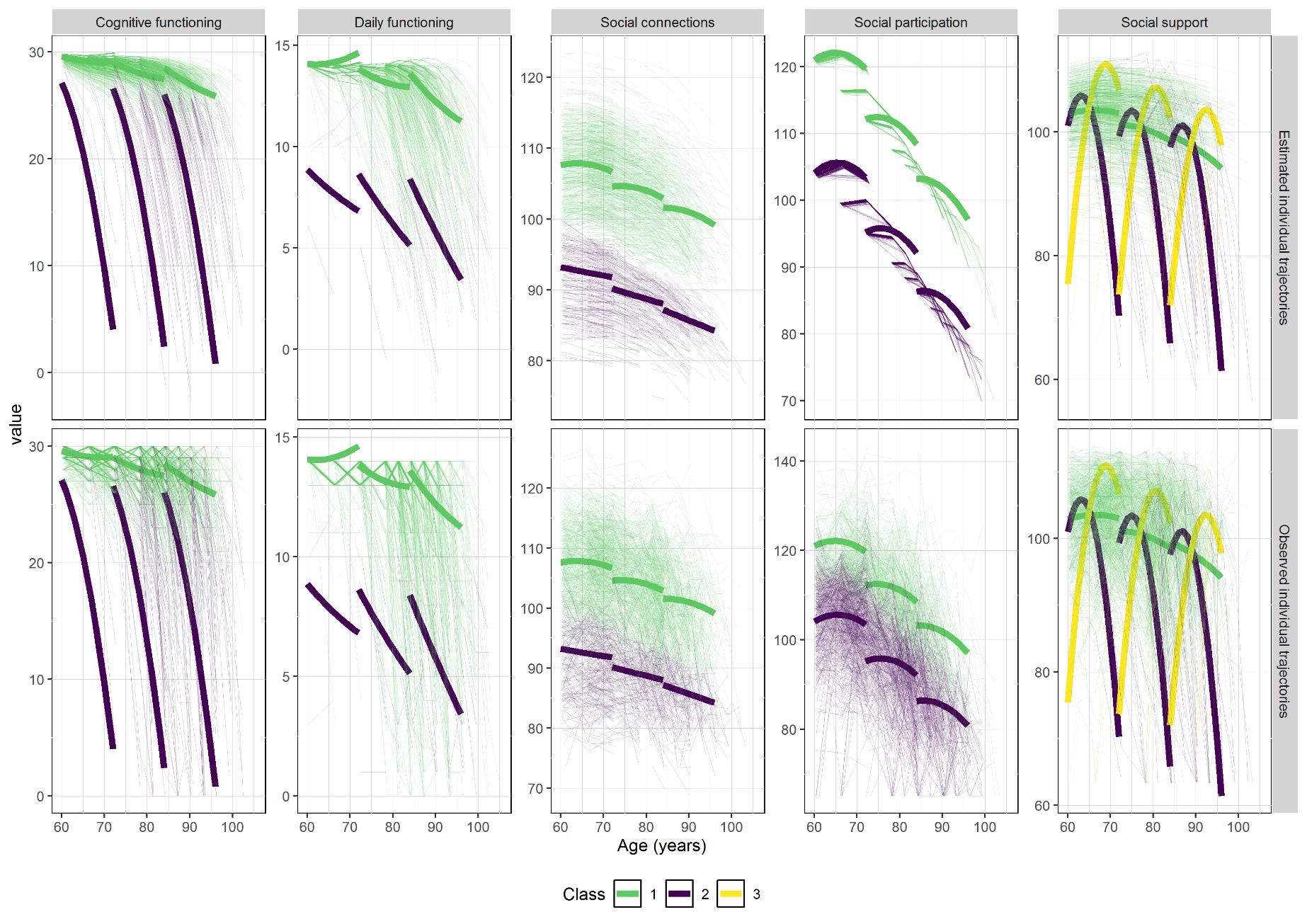
**

**Supplementary Table 4.** Associations of social health indicators trajectories with cognitive and daily functioning concordance and discordance across four groups.

|  | **Concordant** | | **Discordant** | |
| --- | --- | --- | --- | --- |
|  | **High cognitive & daily functioning**  *n* = 2,491 (88.4%) | **Low cognitive & daily functioning**  *n* = 47 (1.7%) | **High cognitive & Low daily functioning**  *n* = 86 (3.0%) | **Low cognitive & High daily functioning**  *n* = 194 (6.9%) |
| **Social participation** |  |  |  |  |
| Class 2 (gradual decline) vs. class 1 (stable), OR (95% CI) ^a^ | Reference | 32.9 (0.02, 51078.4) | 5.3 (2.1, 13.1) | 2.0 (1.3, 3.1) |
| **Social connections** |  |  |  |  |
| Class 2 (initially lower, stable slope) vs. class 1 (stable), OR (95% CI) ^a^ | Reference | 2.0 (1.1, 3.9) | 2.2 (1.3, 3.8) | 1.9 (1.3, 2.7) |
| **Social support** |  |  |  |  |
| Class 2 (declining) vs. class 1 (stable), OR (95% CI) ^a^ | Reference | 0.3 (0, 5081139980899445760) ^b^ | 1.4 (0.3, 6.1) | 2.7 (1.4, 5.0) |
| Class 3 (increasing) vs. class 1 (stable), OR (95% CI) ^a^ | Reference | 6.9 (2.7, 17.4) | 4.5 (2.0, 10.4) | 4.2 (2.3, 7.6) |

*Notes*. OR = odds ratio; CI = confidence interval.

^a^ ORs and 95% CI are based on the pseudoclass method with 20 imputations assigning participants to classes based on their individual class probabilities. The ORs were combined using Rubin's Rule.

^b^ The wide CI is because there were 0 participants in the concordant “low cognitive & daily functioning” group for class 2.
